# Molecular and physiological characterization of two sunflower near-isogenic lines with contrasting haplotypes for leaf senescence

**DOI:** 10.1186/s12870-026-09110-8

**Published:** 2026-06-05

**Authors:** Marco Moroldo, Nicolas Blanchet, Nicolas Pouilly, Stéphanie Pateyron, Marie-Laure Martin, Etienne Delannoy, Cédric Cassan, Yves Gibon, Sébastien Carrère, Guillaume Marti, Nicolas Bernard Langlade

**Affiliations:** 1 UMR LIPME, INRAE, CNRS, Université de Toulouse, Castanet Tolosan, 31326 France; 2 Université Paris-Saclay, CNRS, INRAE, Université Evry, Institute of Plant Sciences Paris-Saclay (IPS2), Gif-sur-Yvette, 91190 France; 3 Université Paris-Saclay, INRAE, AgroParisTech, UMR MIA Paris-Saclay, Palaiseau, 91120 France; 4 UMR BFP, INRAE, Université de Bordeaux, Villenave d’Ornon, 33140 France; 5 Bordeaux Metabolome, MetaboHUB, PHENOME‐EMPHASIS, Villenave d’Ornon, 33140 France; 6 UMR LRSV, MetaToul-AgromiX, CNRS, Université de Toulouse, INP, Auzeville-Tolosane, 31320 France; 7 MetaToul, MetaboHUB, National Infrastructure of Metabolomics and Fluxomics, Toulouse, 31000 France

**Keywords:** Sunflower (Helianthus annuus), Leaf senescence, Quantitative trait locus, Near-isogenic lines, Drought stress, Transcriptome, RNA-seq, Metabolome, Liquid chromatography-mass spectrometry

## Abstract

**Background:**

Leaf senescence is regulated by several genetic mechanisms and environmental factors. During senescence, cellular organelles are degraded and chlorophylls are lost. Concomitantly, macromolecules are broken down into smaller molecules that are transported into tissues like seeds, a process called nutrient remobilization. By reducing photosynthesis and increasing nutrient remobilization, senescence affects seed yield and seed protein. Moreover, its understanding is relevant for agro-ecology, because it could increase nitrogen use efficiency. Sunflower, the fourth oilseed worldwide, is characterized by low water and nitrogen requirements. It shows rapid onset of senescence after anthesis, which hinders its productivity. In this crop, the interaction among senescence, water stress and cover crops for green manure has already been explored from an agronomic perspective. Here, we studied this interaction at the molecular level by comparing morpho-physiological traits and biochemical responses, transcriptome and metabolome of two sunflower near-isogenic lines with contrasting haplotypes for the senescence related LES10.179 QTL. We characterized our plants under water stress and in presence of residues of two cover crops, namely vetch and rye.

**Results:**

The overall response to drought in our findings revealed several novel insights, such as the global decrease of secondary metabolites and the involvement of potentially antioxidant minor compounds. The characterization of the LES10.179 QTL, instead, suggested that senescence was likely controlled by a homolog to *NYC1*, a gene involved in chlorophyll degradation. The LES10.179 QTL also affected seed protein content: the haplotype associated with early senescence lowered it, the other one acting the opposite way. Cover crops had a minor impact on molecular profiles, and no interactive effects were observed between drought and the LES10.179 QTL.

**Conclusions:**

This is the first characterization of a QTL associated with leaf senescence in sunflower. This QTL has also an impact on seed protein content and it is not affected by drought. Therefore, it could prove especially useful for breeding applications. Anyway, further evidence is needed to precisely state whether the observed stay-green phenotype is functional or cosmetic.

**Supplementary Information:**

The online version contains supplementary material available at https://doi.org/10.1186/s12870-026-09110-8.

## Introduction

Leaf senescence is the last stage of leaf development. It is a complex biological process regulated by many genetic layers and influenced by environmental factors such as nutrient deficiency and drought stress [1]. During leaf senescence, pigments are gradually lost and cellular organelles are degraded. In parallel, macromolecules such as proteins, lipids and nucleic acids are broken down into smaller molecules and then are transported into sink tissues, like seeds, a phenomenon defined as nutrient remobilization [2]. The remobilization of nitrogen, also known as nitrogen salvaging, is especially relevant in this context and is tightly linked to the breakdown of chloroplasts. These organelles contain 80% of nitrogen in green leaves, with the carbon-fixing enzyme Rubisco (ribulose-1,5-bisphosphate carboxylase/oxygenase) accounting alone for 10–30% of this element [3].

Another crucial event taking place in senescence is the coordinated degradation of chlorophyll, which is needed because this molecule and its catabolites can cause reactive oxygen species (ROS) burst [4]. After its degradation, chlorophyll itself is not used for nitrogen salvaging, while this is true for chlorophyll-binding proteins [5]. Another category of compounds used for nitrogen remobilization in senescence are nucleobases [6, 7]. Many key genes belonging to the pathways of chlorophyll breakdown and nucleobases salvaging have been identified [5–7], while Rubisco degradation is still poorly understood [8].

Because of the joint occurrence of declining photosynthesis and increasing nutrient remobilization, senescence strongly affects important agronomical traits like seed yield and seed protein. Therefore, it has been widely studied in model plants and crops [2, 9, 10]. Moreover, in the last years this topic has become more relevant in an agro-ecological perspective, because its understanding could facilitate improvements in nitrogen use efficiency and lead to more sustainable agricultural practices [11].

Meanwhile, leaf senescence is also profoundly influenced by environmental factors. Low nitrogen availability tends to accelerate it, while high nitrogen availability can delay it and even reverse it [12, 13]. In turn, delayed senescence can ultimately result in higher yields, as reported in wheat and sunflower [14, 15]. In an agro-ecological context, it has been demonstrated that senescence can be retarded and yields can be improved by increasing nitrogen availability with cover crops (CCs), especially legumes [16, 17]. Similarly, water stress speeds up senescence and decreases yields [18], but recent work in tobacco has also shown that the suppression of leaf senescence leads to increased drought tolerance [19]. Overall, these findings illustrate the complexity of the interplay among senescence, nitrogen availability and water stress.

Sunflower (*Helianthus annuus* L.) is the fourth most important oil crop worldwide. It is known for its low water, nitrogen and pesticide requirements and is adapted to low water-input regimes in warm to semi-arid zones [20]. In sunflower, leaf senescence is triggered abruptly after anthesis. This hinders the capacity of the plant to keep its green leaf area during grain filling and has a strong impact on yield [21]. For this reason, in the last years the molecular bases of leaf senescence in sunflower have been studied through transcriptome and metabolome profiling [21, 22]. Association genetics (GWAS) has also been used [23], but to date no candidate gene has been proposed. Eventually, because of the inherent adaptability of this crop to agro-ecological practices, the interaction among senescence, water stress and cover crops used as green manure has been explored, although only by an agronomical standpoint [24, 25].

In this context, our focus was to compare the morpho-physiological and biochemical traits and the transcriptomic and metabolic profiles of two sunflower near-isogenic lines (NILs) presenting contrasting haplotypes for the leaf-senescence related LES10.179 QTL, and to study the interactive effects among senescence, water stress and cover crops.

The LES10.179 QTL, which had been previously identified through GWAS [23], is located between bases 178,460,000 and 181,257,000 of chromosome 10 (https://www.heliagene.org/HanXRQr2.0-SUNRISE/). One of its haplotypes, derived from the XRQ inbred line, is associated to delayed senescence; a second one, derived from the IR inbred line, is associated to early senescence. These two contrasting haplotypes are incorporated in our two NILs.

Our plants were grown in semi-controlled conditions and characterized under water stress and in presence of residues of two cover crops, i.e. vetch (*Vicia villosa* R.), a legume, and rye (*Secale cereale* L.), a cereal, in the potting medium. We used this experimental setup to test three hypotheses. First, that water stress, the haplotypes of LES10.179, and cover crops may interact statistically, i.e. that each factor may be modified by changes in another factor. Second, that by combining structural genomics and transcriptomics it may be possible to find a putative candidate gene for senescence within the LES10.179 locus. Third, that the genetic mechanisms controlling senescence may also be linked to changes in seed protein content, as described in *Arabidopsis thaliana* [2]. All of these research questions are innovative for the field of sunflower genetics and can contribute to pave new pathways of investigation in the future.

## Methods

### Preparation of the pots

All the steps were carried out on the ‘Heliaphen’ phenotyping facility [26] of the INRAE research center of Auzeville-Tolosane. To mimic the effect of CC incorporation in the soil, 10 L pots were filled with a mix of potting medium (Proveen PAM2, RHP, ‘s-Gravenzande, The Netherlands) and CC residues. The pots were first filled only with potting medium and watered to leach excess nitrogen. Irrigation was applied as follows: (i) three 10-s spray-lance irrigation steps; (ii) four 10-min drip-irrigation steps per day, with a flow rate of 2.7 L/h, for 10 days; (iii) a 10-s spray-lance irrigation step, once per day over the same period of 10 days.

The amount of residues to use for each pot was calculated by considering a theoretical average annual production of CC dry biomass of 3.75 t/ha. Since the surface of each pot was of 0.057 m^2^, this corresponded to 21 gr of dry biomass per pot. The amount of fresh biomass to use was then calculated as fivefold respect to the value of dry biomass (i.e. 105 gr).

CC residues were obtained by cutting CC plots localized at the INRAE experimental farm of Auzeville-Tolosane using a lawn mower on 2022/04/06. They were divided in 105 gr-aliquots and stored at – 20 °C until they were used. Previous to the mixing with the potting medium, residues were thawed at ambient temperature for 15 h. Then, the mixing was performed in three steps: (i) the top 8 cm of the potting medium were removed from each pot and put in a plastic basin; (ii) the amount of potting medium thus obtained was mixed to the crop residues; (iii) the mixture was put back in the pot. The mixing was performed on 2022/07/12, and 72 pots were prepared.

### Plant material and growing conditions

A total of 36 NILs carrying the XRQ-derived allele and 36 NILs carrying the IR-derived allele of the LES10.179 QTL were used for the experiment (Table S01). These plants were selected as described in Document S01. Seeds were germinated in Petri dishes for 48 h at 24 °C. Three germinated seeds were then transplanted to each pot on 2022/07/15 and abundantly irrigated with a spray lance. Water was supplied using drip irrigation, and the plants were thinned out on 2022/08/05 leaving one plant per pot. From 2022/08/05 to 2022/09/02, plants were supplied once a week with 300 mL of a nutrient solution containing 1.2 g/L of Peters Pro Poinsettia Mix 17–07–27 (ICL Growing Solutions, Geldermalsen, The Netherlands) and 0.46 g/L of Hortrilon (Compo, Münster, Germany). Afterwards, from 2022/09/10, plants were irrigated according to one of two different treatments. For control plants (WW), the target relative stress was set at a fraction of transpirable soil water (FTSW) value of 1.0, while for stressed plants FTSW was set at 0.2. Each pot was eventually covered with a 3-mm thick polystyrene sheet to prevent soil water evaporation.

### Measurement of morpho-physiological and biochemical parameters

A total number of 13 physiological and biochemical parameters were measured (Table S02). Height (measured from the base of the stem to the base of the head), stem diameter (measured immediately below the cotyledons) and ratio of senescent leaves were recorded weekly after flowering. Days to flowering (DF) were calculated as the calendar difference between emergence and flowering dates, the latter defined as F1 stage according to BBCH-scale [27] and recorded separately for each single plant. Chlorophyll content, flavonols index and anthocyanins index were measured using a Dualex (Pessl Instruments, Weiz, Austria) on 2022/09/22. Integrated transpired water (ITW) values were calculated as ∫(1-FTSW) as in Rengel et al. [28], and using FTSW values (see previous paragraph) which were recorded daily from 2022/09/09 to 2022/10/21.

Plants were harvested at physiological maturity on 2022/11/02. Aerial parts were recovered and dried at 80 °C for 48 h and plant dry weight, seed dry weight and seed number were recorded. Seed oil content on dry matter was measured with a Minispec MQ10 (Bruker, Billerica, MA, USA). Finally, seed protein content on dry matter was determined as follows. For each plant, 10 mg of dry seeds were ground in liquid nitrogen and fractionated with H₂O/EtOH as previously described [29]. Then, protein was extracted by adding 400 µl NaOH 100 mM to the pellet and by vigorously shaking and heating the tubes at 95 °C for 30 min. After centrifugation at 2,000 g for 10 min, protein in the supernatant was measured using an adaptation of the Lowry method [30]. First, 20 µl of supernatant or bovine serum albumin standard (0 to 2.5 mg/mL in NaOH 0.1 M) were mixed with 120 µl of Biuret reagent (Sigma Aldrich, Saint Louis, MO, USA) and incubated for 15 min in a flat-bottom microplate at room temperature. Then, 15 µl of Folin-Ciocalteu reagent (Sigma Aldrich) were added. After incubation at room temperature for 30 min, absorbance was read at 630 nm in a MP96 microplate reader (SAFAS, Monaco).

Five samples presented missing values for some of the recorded parameters and were discarded. Therefore, the final data set for physiological and biochemical parameters included 67 samples.

### Analysis of morpho-physiological and biochemical parameters

The “prcomp” function of the ‘stats’ R package v 4.2.2 was used to perform a scaled PCA using all the morpho-physiological and biochemical parameters. ANOVA was performed by fitting, on each parameter, a linear model that included the three factors under study, i.e. water stress, allelic states of the LES10.179 QTL and cover crops, and their interactions. The ‘aov’ function was used for the modeling step, while the ‘emmeans’ and ‘pairs’ functions of the ‘emmeans’ R package v 1.10.4 were used to compute estimated marginal means and to perform t-tests on pairwise comparisons. Because the results indicated no second order and third order interactions, a second linear model was fit on the data, this time taking into account only single factors. Finally, *p*-values were Benjamini–Hochberg adjusted for multiple comparisons using the ‘p.adjust’ R function and a threshold of 0.05 was imposed.

### Sample preparation and transcriptome primary analysis

For each plant, the 5th and the 6th leaves from the top were harvested without petiole, immediately frozen in liquid nitrogen and stored at – 80 °C until they were used. The sampling was performed on 2022/09/19 from 10:45 to 11:25 (CET). Frozen samples were ground using a ZM 200 grinder (Retsch, Haan, Germany) with a 0.5-mm stainless steel sieve.

For each sample, 100 mg of frozen powder were treated with the NucleoSpin RNA II kit (Macherey–Nagel, Düren, Germany). The concentrations and the 260/280 ratios of the RNAs were determined using a Nanodrop (Thermo Fisher Scientific, Waltham, MA, USA), while their integrity was checked using an Agilent 2100 Bioanalyzer (Agilent, Santa Clara, CA, USA) and an Agilent RNA 6000 Nano Chip (Agilent). RIN values ranged from 7.4 to 8.3. The Illumina Stranded mRNA Prep Kit (Illumina, San Diego, CA, USA) was used to prepare the sequencing libraries. Paired-end 150 bp sequencing was performed using a NovaSeq 6000 (Illumina) by the POPS sequencing facility (INRAE research center of Saclay). Eventually, FASTQ files were submitted to the SRA repository (www.ncbi.nlm.nih.gov/sra) under the BioProject name PRJNA1321532.

Raw paired-end reads were processed with the nf-core/rnaseq v 3.0 (https://doi.org/10.5281/zenodo.4323183) pipeline, and aligned to the sunflower reference genome ‘HanXRQr2.0’ (GCA_002127325.2) using Salmon v 1.4.0. Read counts were retrieved at the gene level. After a PCA-based quality control, one WW sample appeared to cluster with WS samples. It was considered as an outlier and was discarded.

All the subsequent steps of data pre-processing, normalization and quality control were performed with the ‘DiCoExpress’ R script-based tool v2 (https://forgemia.inra.fr/GNet/dicoexpress) and using default parameters. The obtained ‘expr’ expression matrix was used to perform a scaled PCA analysis using the ‘prcomp’ function of the ‘stats’ R package v 4.2.2.

### Differential analysis

Differential analysis was also carried out using ‘DiCoExpress’, which fits a generalized linear model (GLM) for each gene. Because ‘DiCoExpress’ cannot handle three-factor experimental designs, we first defined a mock variable by collapsing the allelic states of the LES10.179 QTL and the cover crops, and we then fitted a GLM including water stress, the mock variable and their interaction. The obtained results indicated that cover crops did not influence the model. Therefore, we fit a second GLM by considering this time the effects of water stress, the allelic states of the LES10.179 QTL and their interaction. Finally, because no interaction between the two factors was found, we fit a third GLM by taking into account only the single effects of water stress and the allelic states of the LES10.179 QTL and the two factors were tested (‘WS contrast’ and ‘LES10.179 contrast’). A threshold of 0.05 for Benjamini-Hochberg-adjusted *p*-values was used in all the cases.

### Definition of a list of sunflower drought markers

To define a list of sunflower drought markers, we first retrieved the protein sequences of 17 *A. thaliana* genes (Table S10) considered predictive of hydric stress [31]. Then, the selected protein sequences were used to perform a tBLASTn analysis and the corresponding sunflower hits were filtered by keeping those with an identity value higher than 50% and an alignment length higher than 100 amino acids. If a protein aligned to the same sunflower hit in multiple regions, and each time the alignment length was lower than 100 amino acids, the hit was kept if the sum of the alignment lengths of the different regions was higher than 100 amino acids and at least 50% of the regions had an identity value higher than 50%. Eventually, 90 potential unique sunflower drought markers were thus obtained (Table S10).

### Evaluation of the impact of residual heterozygosity on differential analysis

To take into account the potential impact of the regions of residual heterozygosity on the results of the ‘LES10.179 contrast’, the genomic positions of all of the heterozygous Axiom markers found outside the LES10.179 QTL region in the F6 individual used to produce the plants used in this work were retrieved. Then, for each marker, an interval was defined by adding 50,000 bp downstream and upstream respect to the position of the marker itself. These intervals were intersected with a .gtf file bearing the genomic positions of all the differentially expressed genes (DEGs) using the ‘intersect’ function of ‘BEDTools’ [32]. Finally, the thus obtained overlapping regions were visually inspected and only those larger than 20,000 bp and presenting at least 50% of heterozygous markers were considered reliable and kept. This allowed to identify four regions of residual heterozygosity spanning 4.07 Mb and containing 27 genes, which were not considered for the downstream steps of analysis.

### Enrichment analysis

Enrichment analysis was performed as follows. First, each of the two DEG lists obtained from the ‘WS contrast’ and the ‘LES10.179 contrast’ was split in two sub-lists corresponding to the genes with positive and negative log2FC values. Then, the four sub-lists were analyzed with ClueGO v 2.5.8 [33]. A right-sided test was used to highlight enriched pathways and significance was set at a Benjamini-Hochberg-adjusted *p*-value of 0.01. Two custom ontology terms were used, i.e. (1) a KEGG term, and (2) a ROS-specific term based on Willems et al. [34]. These two terms were built as described in Leconte et al. [35].

### Definition of a list of sunflower senescence associated genes (SAGs)

To define a list of sunflower SAGs, the sequences of the 5,094 *A. thaliana* proteins found at the ‘Leaf Senescence Data Base’ (https://ngdc.cncb.ac.cn/lsd/) as of June 2025 were retrieved and used to perform a tBLASTn analysis. Sunflower hits were filtered as already described for drought markers, and 8,373 potential unique sunflower SAGs were obtained (Table S11).

### Characterization of the LES10.179 QTL genomic region

The genes found in the LES10.179 QTL region of the XRQ-derived haplotype were extracted from the ‘Heliagene’ website (https://www.heliagene.org/) and used to perform a synteny analysis as described in Document S02.

### Metabolome analysis

For each sample, 700 µL of MTBE/MeOH (75:25) were added to 100 mg of frozen leaf powder obtained as previously described and the mix was vortexed for 30 s. The sample was then sonicated for 3 min and 700 µL of H₂O/MeOH (75:25) were added. Subsequently, each tube was centrifuged at 14,000 rpm for 3 min at 5 °C. Then, the lower hydrophilic phase was recovered and dried with a SpeedVac. The dry mass was weighed and resuspended in MeOH/H₂O (80:20). QC samples were created by pooling a small volume of each of the samples. Because of technical issues, three samples could not be processed. Therefore, the data set for metabolome analyses included 69 samples.

Ultra-high-performance liquid chromatography high-resolution mass spectrometry (UHPLC-HRMS) was performed on a Q Exactive Plus Quadrupole-Orbitrap (Thermo Fisher Scientific, Waltham, MA, USA) mass spectrometer, equipped with a heated electrospray probe (HESI-II) coupled to a UHPLC Vanquish H system (Thermo Fisher Scientific). HILIC chromatography was performed using a Poroshell 120 HILIC-Z column (2.7 µm, 2.1 × 150 mm, Agilent) equipped with a guard column. The mobile phase A (MPA) was 0.1% formic acid and 10 mM ammonium formate in water, and the mobile phase B (MPB) was 0.1% formic acid and 10 mM ammonium formate in acetonitrile. The solvent gradient was 10% MPA (0—0.5 min), 10% MPA to 50% MPA (0.5—9 min), 50% MPA (9—11 min), 10% MPA (11.1—20 min). The flow rate was 0.3 mL/min, the column temperature was set to 40 °C, the autosampler temperature was set to 5 °C and the injection volume was fixed to 1 μL.

Mass detection was operated in positive ionization (PI) and negative ionization (NI) mode with a resolving power of 35,000 (full width at half-maximum (FWHM) at 400 m/z) for MS1 and a resolving power of 17,500 for MS2, with an automatic gain control (AGC) target of 1 × 106 for full scan MS1 and 1 × 105 for MS2. Ionization spray voltages were set to 3.5 kV for PI and 2.6 kV for NI, and the capillary temperature was kept at 256 °C. The mass scanning range was 100–1500 m/z. Each full MS1 scan was followed by data-dependent acquisition of MS2 spectra for the four most intense ions using stepped normalized collision energy of 20, 40, and 60 eV.

UHPLC-HRMS raw data were processed with MS-DIAL v 5.1 [36] for mass signal extraction between 100 Da and 1,500 Da from 0.5 min to 12 min. MS1 and MS2 tolerance values were set to 0.01 Da and 0.05 Da in centroid mode, respectively. The optimized detection threshold was set to 1 × 105 for MS1 and 10 for MS2. Peaks were aligned on a quality control reference file with a retention time tolerance of 0.15 min and a mass tolerance of 0.015 Da.

The MS-DIAL peak list was filtered with MS-CleanR v 1.0 [37] by selecting all filters with a minimum blank ratio set to 0.8, a maximum relative standard deviation (RSD) set to 40, and a relative mass defect (RMD) ranging from 50 to 3,000. Peak annotation was performed using an in-house database from the MetaToul-AgromiX facility for annotation level ‘1’ and the FragHub database v6 [38] for annotation level ‘2a’ (reverse dot product > 850), ‘2b’ (reverse dot product > 700) and level ‘4’, i.e. MS/MS analog [39]. The remaining features were annotated with MS-FINDER v 3.61 [36]. MS1 and MS2 tolerances were set to 5 ppm and 10 ppm, respectively. Formula finder was only processed with C, H, O, N, P, and S atoms. Databases based on the genus *Helianthus* and the family Asteraceae were built using the Dictionary of Natural Products (DNP on DVD v. 30.1, CRC Press) for annotation level ‘3a’. Additionally, internal generic MS-FINDER databases were used for annotation level ‘3’. Annotation was performed prioritizing genus- and family-specific databases and then generic ones, using an internal Knime based workflow [40].

All the 2,581 identified metabolites (Table S08) where used to perform a scaled PCA using the ‘prcomp’ function of the ‘stats’ R package v 4.2.2. Differentially abundant metabolites (DAMs) were identified by fitting, on each molecule, a linear model that included the three factors under study, i.e. water stress, the allelic states of the LES10.179 QTL and cover crops, and their interactions. The ‘aov’ function was used for the modeling step, and the ‘emmeans’ and ‘pairs’ functions of the ‘emmeans’ R package v 1.10.4 to compute estimated marginal means and perform t-tests on pairwise comparisons. Since the results indicated no interaction between the factors, a second linear model was fit without considering interactions. Then, *p*-values were Benjamini–Hochberg adjusted for multiple comparisons using the ‘p.adjust’ R function and a threshold of 0.05 was imposed. DAMs with an annotation level higher than ‘3a’ were kept for the downstream analyses and manually re-annotated using CID codes.

## Results

### Analysis of morpho-physiological and biochemical parameters

**Fig. 1:**
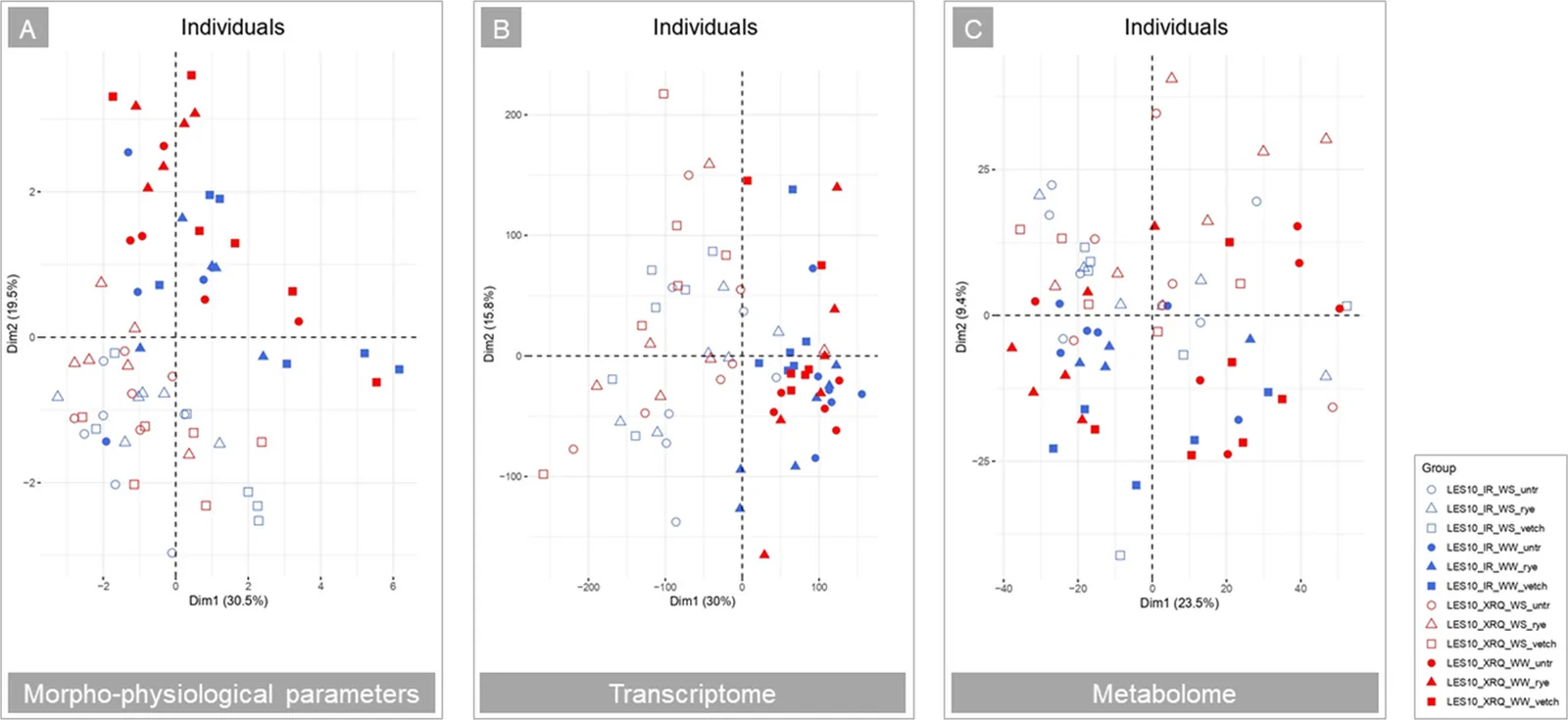
Individual plots of the first two components of the PCA analyses obtained using the three available data sets. **A** PCA run on morpho-physiological parameters; **B** PCA run on transcriptome data; **C** PCA run on metabolome data. In all of the three cases the samples are colored according to their biological group, and biological groups are defined as all the possible combinations among the three factors under study, namely water status, the haplotypes of the LES 10.179 QTL and the effect of cover crops residues mixed to the potting medium used to grow the plants (see legend)

A total number of 13 morpho-physiological and biochemical parameters were available for 67 samples (Table S01, Table S02). PCA separated water stressed (WS) and control samples (WW) in a clear way, while the two haplotypes of the LES10.179 QTL and the cover crops did not have any impact on the structure of the data (Fig. 1A).

**Fig. 2:**
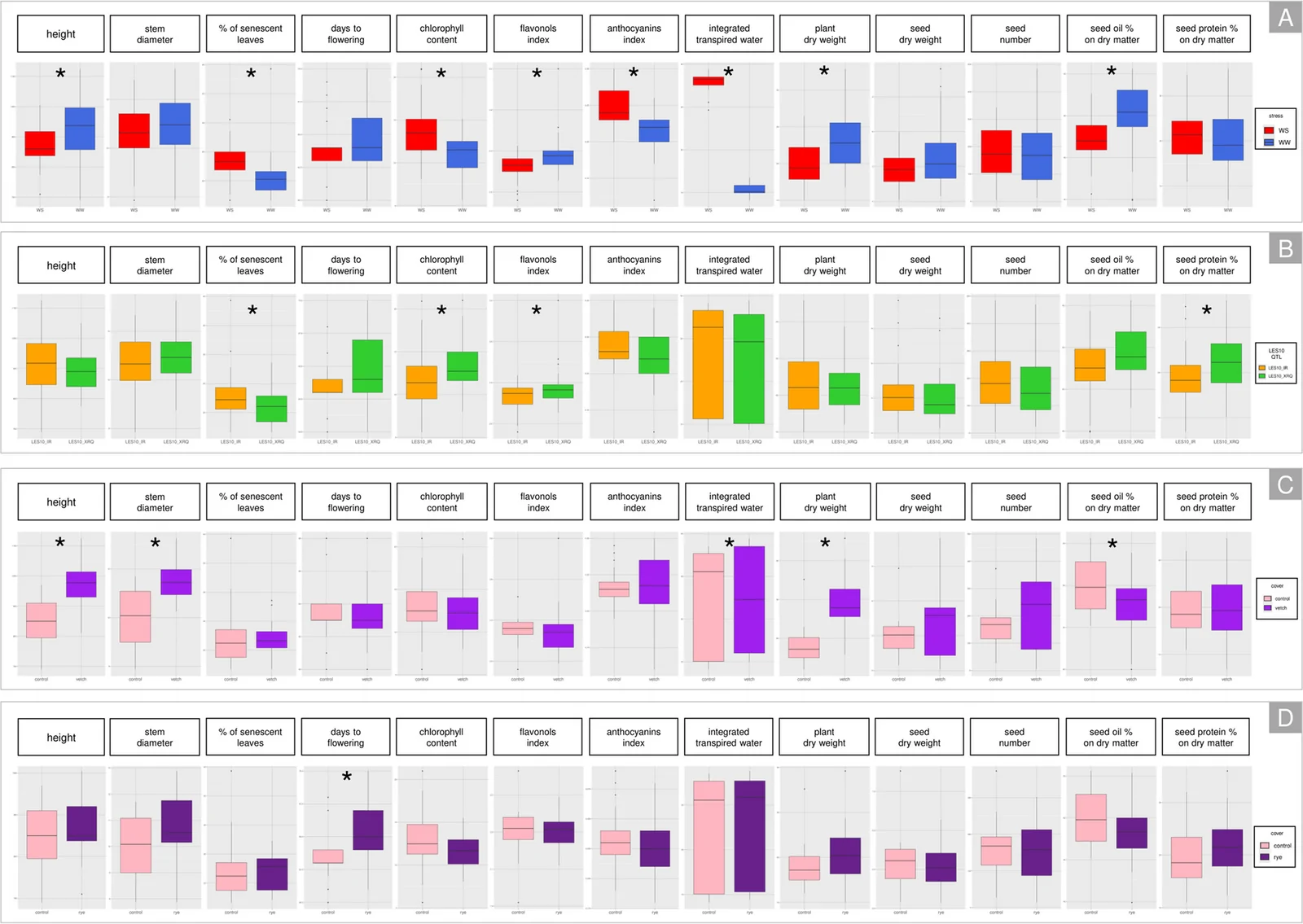
Boxplots of the 13 morpho-physiological and biochemical parameters measured in our project. For ease of reading, the names of some parameters have been shortened. **A** Boxplots of the 13 parameters obtained by comparing water stressed samples to controls; **B** Boxplots of the 13 parameters obtained by comparing samples carrying the XRQ-derived haplotype of the LES10.179 QTL to samples carrying the IR-derived one; **C** Boxplots of the 13 parameters obtained by comparing samples grown in presence of residues of vetch in the potting medium to controls; **D** Boxplots of the 13 parameters obtained by comparing samples grown in presence of residues of rye in the potting medium to controls. In all the cases, the asterisks indicate adjusted *p*-values < 0.05 for post-hoc t-student tests

ANOVA showed that WS had the strongest influence among the three factors under study, since it significantly influenced eight parameters. Among the most relevant traits, height, plant weight and flavonols index were decreased by stress, whilst chlorophyll content and the ratio of senescent leaves were increased. The LES10.179 QTL, instead, influenced only four parameters. As expected, the ratio of senescent leaves was decreased in the late-senescence XRQ-derived haplotype, while chlorophyll content and flavonols index were increased. Interestingly, the same haplotype also presented a higher value of seed protein content on dry matter (Table S03, Fig. 2). Finally, vetch residues affected five parameters and rye residues just one. Consistently with our recent findings [25], in the case of CCs, the parameters measured during the vegetative stage were more affected than those measured after flowering. Indeed, the exception to this pattern was represented by seed oil content on dry matter (Table S03, Fig. 2).

### Transcriptome profiling

RNA-seq was performed on all of the 72 leaf samples (Table S01). Sequencing libraries had an average size of 76.1 M reads and the average ratio of aligned reads was 89%, i.e. 67.8 M reads per sample (Table S04). After the removal of an outlier, the downstream steps of analysis were carried out on the remaining 71 samples. As in the case of morpho-physiological parameters, PCA split the samples according to the level of water stress and not according to the two other factors under study (Fig. 1B).

CCs did not influence gene expression, and there were no statistically significant interactive effects among water stress and the haplotypes of the LES10.179 QTL (see methods). Therefore, differential analysis (DA) was based only on water stress and the allelic states of our QTL, and was carried out by performing two contrasts, i.e. the first one comparing water stressed to control samples (‘WS contrast’) and the second one comparing samples carrying the XRQ-derived haplotype of the LES10.179 QTL to samples carrying the IR-derived one (‘LES10.179 contrast’).

**Fig. 3:**
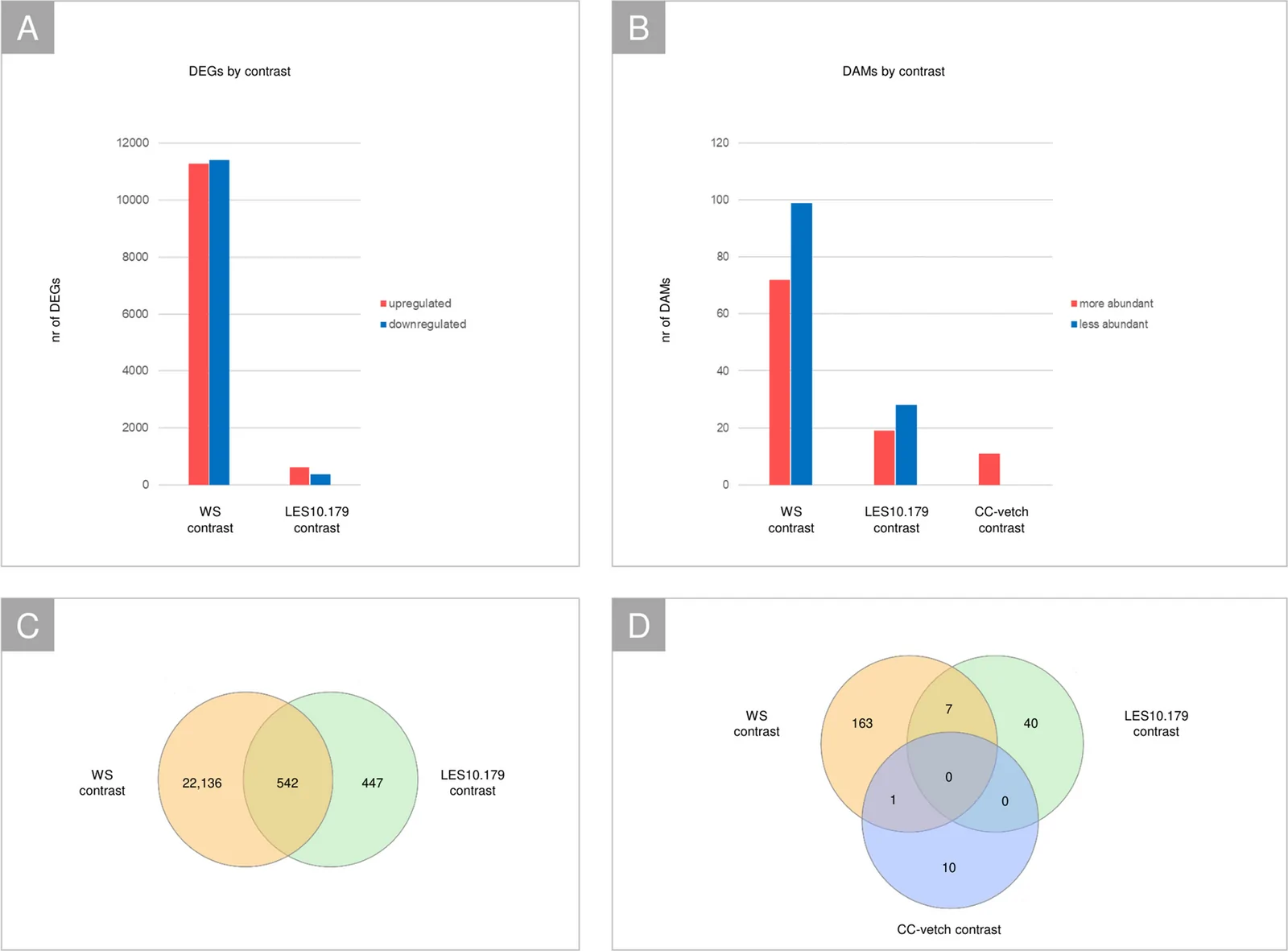
Statistics relative to differential expression analysis performed on transcriptome and differential abundance analysis performed on metabolome. **A** Barplots showing upregulated and downregulated DEGs for the ‘WS contrast’ and for the ‘LES10.179 contrast’; **B** Barplots showing more abundant and less abundant DAMs for the ‘WS contrast’, for the ‘LES10.179’ contrast and for the contrast among samples grown in presence of vetch residues in the potting medium and controls; **C** Venn diagram obtained by comparing DEGs resulting from the ‘WS contrast’ and from the ‘LES10.179 contrast’; **D** Venn diagram obtained by comparing DAMs resulting from the ‘WS contrast’, the ‘LES10.179 QTL’ contrast, and the contrast among samples grown in presence of residues of vetch in the potting medium and controls

The ‘WS contrast’ yielded 22,678 DEGs, of which 11,271 were upregulated and 11,407 downregulated (Table S05A, Fig. 3). Almost a third of the 50 top upregulated genes of the list was unannotated, and only a few could be related to the drought response, like the expansin *HanXRQr2_Chr16g0744161*. Nonetheless, considering the whole ‘WS contrast’ list, we identified 48 putative homologs to markers of a physiologically relevant hydric stress [31], a significant enrichment when using the ‘expr’ matrix as a background (see methods). As expected, most of these transcripts (i.e. 30 out of 48) were overaccumulated under stress (Table S05A).

Among these markers, there was *HanXRQr2_Chr16g0775841,* a homolog to the well-described *RAB18* dehydrin. Dehydrins play a relevant role in the response to abiotic stresses [41] and in sunflower they seem to overaccumulate in a consistent way under drought [42–45]. Noteworthy, four other *RAB18* homologs were present in the ‘WS contrast’, but were not explicitly included in our set of sunflower drought markers (see methods) because of the tBLASTn thresholds imposed to assign homologies.

To gather more insights into the mechanisms underlying the response to drought, we ran an enrichment analysis using ClueGO [33]. This analysis returned 28 upregulated pathways, among which some were related to glycolysis, amino acid biosynthesis, proteasome activity and ROS metabolism (Table S06A). Other 19 pathways, instead, were downregulated, including some processes linked to photosynthesis, secondary metabolite biosynthesis, amino acid biosynthesis and sugar metabolism (Table S06A).

The ‘LES10.179 contrast’ yielded 1,016 DEGs, of which 27 co-localized with regions of residual heterozygosity. These 27 genes were discarded, and the remaining 989 were used for the downstream analyses (Table S05B). Among these genes, 609 were upregulated and 380 were downregulated (Fig. 3). Enrichment analysis returned one upregulated term, i.e. ‘metabolism of xenobiotics by cytochrome P450’, and two downregulated ones, i.e. ‘pyrimidine metabolism’ and ‘sulfur metabolism’ (Table S06B). In total, 126 genes appeared to be potential senescence-associated genes or SAGs (see methods). Interestingly, DEGs presented a particularly skewed genomic distribution, with 71 of them mapping on the 2.8-Mb genomic interval on chromosome 10 (178,460,000–181,257,000) corresponding to the LES10.179 QTL region.

No statistically significant interactive effects were found between water stress and the LES10.179 QTL. Conversely, the intersection between the ‘WS contrast’ and the ‘LES10.179 contrast’ lists yielded 542 shared DEGs. This set of genes did not present any specific ontology enrichment. It did not include any drought marker, while it contained 53 out the 71 DEGs that mapped on the LES10.179 QTL region.

### Characterization of the LES10.179 genomic region

Because of the peculiar distribution of the DEGs associated to the ‘LES10.179 contrast’, the LES10.179 QTL genomic region was further investigated by looking at the patterns of synteny between the XRQ-derived and the IR-derived haplotype. The LES10.179 QTL is located at one of the telomeres of chromosome 10 and shows, accordingly, high gene density. Indeed, this interval contains 153 protein-coding genes in the former haplotype and 148 in the latter, with 19 cases of gene presence/absence variation (Table S07).

**Fig. 4:**
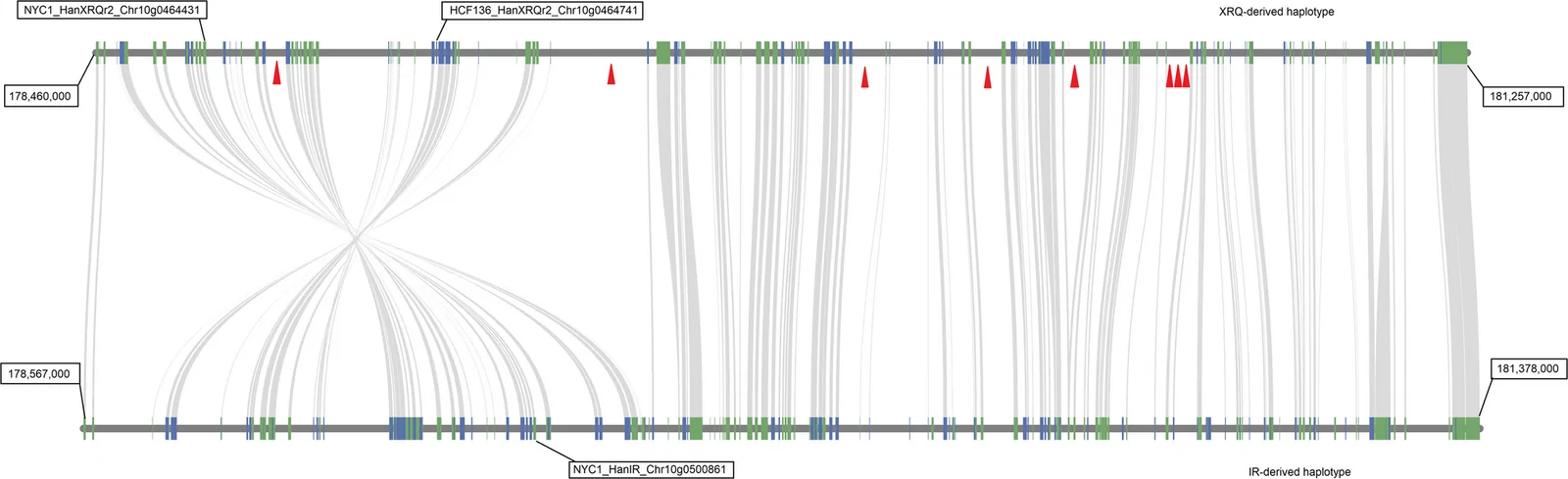
Plot showing the gene-level synteny between the XRQ-derived and the IR-derived haplotypes of the LES10.179 QTL. The two homologs of the *NYC1* gene and the homolog of the *HCF136* gene, which is present only in the XRQ-derived haplotype, are located in the inverted region and are indicated in boxes. The SNPs associated to senescence progression speed and used to define the LES10.179 QTL are indicated by red triangles. The chromosome positions correspond to the approximate start of the first gene and to the approximate end of the last gene belonging to the LES10.179 QTL

Interestingly, synteny analysis identified an 873-kb inversion between bases 178,540,000 and 179,413,000 of the XRQ-derived haplotype (Fig. 4). This region included 49 genes in the XRQ-derived haplotype and 43 in the IR-derived haplotype, corresponding to six gene insertions in the former. Among these 49 genes, the most relevant in the context of senescence was *HanXRQr2_Chr10g0464431*, a homolog to the *NYC1* chlorophyll b reductase that was also annotated as a SAG. The genes located outside the inverted region, instead, were more elusive in terms of biological functions.

### Metabolome profiling

The metabolome of 69 samples (Tables S01 and S08) was analyzed in an untargeted way using a liquid chromatography mass spectrometry (LC–MS) approach in hydrophilic interaction chromatography (HILIC) mode. After the identification of an outlier, the downstream analysis steps were performed on the remaining 68 samples. A PCA individual plot (Fig. 1C) separated the samples according to stress, but not according to the haplotypes of the LES10.179 QTL or CCs.

As in the case of transcriptome, ANOVA did not detect interactions among the factors under study, and thus only the separate effect of each factor was considered. The comparison among WS and WW samples identified 171 DAMs (Table S09A). Despite the differences in the experimental setups and in the analysis workflows, the overall composition was similar to what had already been observed in a similar context in sunflower [46, 47], but with a higher proportion of amino acids (AAs) and a lower proportion of flavonoids. This could be due to the fact that HILIC tends to retain moderate to highly polar compounds more than the C18 columns used in the previous works [48].

Water stress increased the abundance of the large majority of amino acids and carbohydrates, the latter mostly represented by sugars in our samples. Conversely, organic acids mainly decreased, as well as most secondary metabolites, i.e. hydroxycinnamic acids, flavonoids, monolignols, lignans and terpenes. Phenolic acids, instead, were generally more abundant. Concerning the remaining groups of compounds of interest, dimethylsulfoniopropionate (DMSP) was more abundant, while most vitamers of the identified vitamins (i.e. B5, B6, B10 and dehydroascorbate, an oxidized form of vitamin C) decreased.

The comparison of the two haplotypes of the LES10.179 QTL yielded 47 DAMs, whose overall composition presented some analogies with the one observed under drought (Table S09B). The majority of secondary metabolites, i.e. hydroxycinnamic acids, flavonoids and terpenes decreased. Phenolic acids, instead, as well as AAs and carboxylic acids, did not show a clear pattern. Uracil, a nucleobase of interest in the context of nitrogen salvage, was more abundant in the XRQ-derived haplotype.

Finally, the comparison between the samples grown with nitrogen-rich residues of vetch and controls yielded 11 differentially abundant metabolites, mainly terpenes and hydroxycinnamic acids (Table S09C). All of these molecules were more abundant in treated samples. Rye residues, instead, did not induce any change in the composition of metabolome.

## Discussion

### Drought increases osmolytes and decreases secondary metabolites in the LES10.179 NILs

As an introductory remark, morpho-physiological parameters suggested a proper implementation of water stress. Plant weight, plant height and oil content decreased in stressing conditions, while the percentage of senescent leaves and ITW increased (Table S03, Fig. 2), which agreed with the literature [28, 49]. In contrast, anyway, seed number and seed weight were not affected. Importantly, the implementation of WS was confirmed by the expression of specific molecular markers [31].

The overall molecular response to water deficit in our NILs was similar to that described in other sunflower lines [44, 50]. Anyway, the joint analysis of transcriptome and metabolome, the large number of biological replicates (36 WS and 35 WW samples for transcriptome and 35 WS and 33 WW samples for metabolome) and the specific LC–MS protocol used for metabolomics highlighted several relevant and previously undescribed aspects. In this regard, a meaningful example was constituted by the pathways involved into the biosynthesis of the different classes of metabolites, i.e. amino acids, carbohydrates, organic acids, secondary metabolites and some minor compounds.

Amino acids typically accumulate in water-stressed plants, as reported in sunflower [47] and other species like arabidopsis [51], poplar [52] and sesame [53]. This increase affects osmotically active AAs like proline, aromatic AAs needed to synthesize secondary metabolites (e.g. phenylalanine) and others such as serine [51]. All of these AAs, as well as some of their reaction intermediates, increased in WS (Table S09A), and many genes in the corresponding pathways were upregulated (Table S05A).

Among all the identified amino acids, the most relevant case was represented by proline, which was, as expected, more abundant under stress (log2FC = 1.3). The accumulation of this AA as a response to water scarcity has been extensively shown in sunflower [50, 54, 55], poplar [52], pea [56], sesame [53], asparagus [57] and, interestingly, many desert species [58]. The higher level of proline was matched by the enhanced expression of four homologs of the delta-1-pyrroline-5-carboxylate synthase *P5CS1* (Table S05A), which is the rate-limiting enzyme of the corresponding biosynthetic pathway and has already been described in sunflower [28, 59] and other plants [60].

The increase of proline in WS was also consistent with the expression patterns of other DEGs, like *HanXRQr2_Chr17g0823771*, an ornithine aminotransferase participating in the anabolism of the molecule [61] which was activated (log2FC = 0.9), and two proline dehydrogenases, *HanXRQr2_Chr15g0712341* and *HanXRQr2_Chr12g0559381*, which are instead involved in the catabolic steps and which were inhibited.

Carbohydrates are another important class of metabolites involved in osmotic adjustment, with a prominent role of sugars and polyols. In our experiment only sugars were detected, almost all of which increased under stress. Some of them, namely fructose, mannose, melibiose and melezitose, are known to accumulate under drought in species such as arabidopsis, tea plant and sesame [53, 62, 63]. Our findings were also consistent with Moschen and collaborators [55], although the identified sugars differed, and were confirmed by enrichment analysis (Table S06A). Interestingly, the ‘sucrose and starch metabolism’ pathway was downregulated in WS (Table S06A), suggesting that carbon export and storage were decreased to promote osmolyte accumulation.

Organic acids present a more complex picture. Most of them are involved in the citric acid (TCA) cycle and are increased by drought, although not systematically [62] and, in some species, only in drought-tolerant genotypes [57]. Other molecules of this class, instead, are precursors of AAs and other compounds. Indeed, the majority of the organic acids detected in our case were not TCA cycle-related and were lower in WS. Succinic acid, a key intermediate of the TCA cycle, was instead higher (log2FC = 2.5), suggesting activation of this process as previously shown [55]. Enrichment analysis confirmed that some TCA cycle-related pathways were activated and other ones inhibited (Table S06A).

Contrarily to what observed for primary metabolites, all the main groups of secondary metabolites were less abundant in WS, except for phenolic acids (Table S09A). This was paralleled by the inhibition of many genes involved for instance in flavonoid biosynthesis, like the anthocyanin malonyltransferase *HanXRQr2_Chr17g0808551* and the chalcone synthase *HanXRQr2_Chr11g0470821,* or implicated in terpene biosynthesis, like the beta-caryophyllene synthase *HanXRQr2_Chr02g0058571* and the R-linalool synthase *HanXRQr2_Chr03g0105031* (Table S05A). The results of overrepresentation analysis (Table S06A) and the decreased values of flavonols index measured by Dualex also mirrored these findings (Table S03, Fig. 2), and were also consistent with previous works in sunflower showing inhibition of the biosynthesis of terpenes [64] and of secondary metabolites in general [59] under drought. It is to note, anyway, that the anthocyanins index was higher in WS (Table S03, Fig. 2).

Generally speaking, secondary metabolites mainly act as antioxidants under abiotic stress and, therefore, tend to accumulate in this condition [65]. This has been shown, for instance, for flavonoids [66–68], carotenoids [69] and terpenes [70, 71]. However, more complex patterns have also been described. In *Ligularia fischeri*, a plant of the Asteraceae family, WS reduces hydroxycinnamic acids and flavonols and increases flavones and anthocyanins [72]. In *Lavanda angustifolia*, terpenes accumulate in drought-tolerant genotypes and diminish in sensitive ones. Assuming an antioxidative role of these molecules, it could be speculated that sensitive varieties trade-off between growth and defense, while tolerant ones activate their response mechanisms while keeping their growth levels [73].

This could therefore suggest that our NILs behave more like sensitive genotypes than like tolerant ones, even if more evidence is needed to support this. It is also to remark that, in our case, phenolic acids are the only secondary metabolites accumulating in WS, a pattern observed in other Asteraceae such as *Achillea pachycephala* [74] and *Tagetes* spp. [75].

Finally, some minor compounds appeared of interest because of their potential antioxidant activity. The first example was dehydroascorbate, which decreased under WS (log2FC = −0.7). Dehydroascorbate is the major oxidized form of ascorbate, a key reducing agent in the ascorbate–glutathione cycle [62]. Usually accumulated in WS, it has been found decreased under dehydration [76]. Three homologs to the *DHAR* dehydroascorbate reductase*,* which converts dehydroascorbate to ascorbate, were inhibited in our data (Table S05A). Pyridoxal and pyridoxine, two vitamers of vitamin B6, were detected under WS (log2FC = −0.5 and log2FC = 0.6). Vitamin B6 has antioxidant capacities [77] and pyridoxine increases under drought [78]. Another intriguing case was dimethylsulfoniopropionate (DMSP), an organosulfur compound which accumulates under water stress [79, 80]. DMSP was higher in WS (log2FC = 0.6) and some genes potentially related to its anabolism were activated (Table S05A).

When looking at the pathways not related to metabolite biosynthesis, our results mainly agreed with the literature. Several photosynthesis-related terms were inhibited by water stress (Table S06A), as already shown in sunflower [59, 64] and other species [81]. At the same time, higher levels of chlorophyll were found (Table S03, Fig. 2). This could seem inconsistent with the inhibition of photosynthetic pathways and with the fact that drought reduces chlorophyll in most species [82], but it has to be noted that several exceptions to this general pattern exist. In soybean and wheat, for instance, increased concentrations of chlorophyll have been detected in drought-tolerant genotypes [83, 84].

Contrarily, the pathways related to the ubiquitin–proteasome system were activated under WS (Table S06A), with a large number of upregulated key genes such as for instance *HanXRQr2_Chr16g0732101*, a ubiquitin-activating enzyme, *HanXRQr2_Chr10g0449461*, a ubiquitin-conjugating enzyme, and *HanXRQr2_Chr13g0565711*, a ubiquitin-protein ligase [85]. Proteolysis plays many roles in drought stress, including the degradation of damaged proteins and the remobilization of AAs [86]. Peptides produced by proteolysis can also regulate the production of ROS [87]. Indeed, three pathways related to ROS metabolism were influenced by WS (Table S06A).

Eventually, the activation of the ethylene signaling pathway was suggested by the upregulation of *HanXRQr2_Chr09g0395901*, *HanXRQr2_Chr17g0820271* and *HanXRQr2_Chr17g0779421*, three homologs to the *ACS* 1-aminocyclopropane-1-carboxylate synthase. *ACS* is the rate-limiting enzyme in the biosynthesis of ethylene and its implication in WS is well characterized in several species [88, 89].

### The LES10.179 QTL potentially controls leaf senescence by acting on chlorophyll degradation

Respect to what observed for WS, the molecular profiles associated to the LES10.179 QTL were less clear-cut. Moreover, comparing our findings to those previously obtained in sunflower [21] was not straightforward due to relevant differences in the experimental setups and in the analysis workflows.

At the transcriptome level, overrepresentation analysis yielded only three enriched pathways (Table S06B). The upregulated term ‘metabolism of xenobiotics by cytochrome P450’ could be artifactual, since three of the four associated genes were glutathione transferases, whose role in xenobiotic metabolism is disputed and which could rather be linked to ROS metabolism [90]. Indeed, antioxidant systems activate in senescence because chlorophyll causes oxidative burst [4, 91].

The downregulated term ‘pyrimidine metabolism’, instead, could suggest a deceleration of pyrimidine salvage, a process that allows the recycling of nitrogen from pyrimidines [92], in the XRQ-derived haplotype. This could be consistent with delayed senescence and with the higher levels of uracil observed (Table S09B). While the enrichment of this pathway was not found by Moschen et al. [21], the same authors detected the accumulation of asparagine in the early-senescence line used in their study, pointing to the activation of nitrogen salvaging [21, 93]. Thus, even if the identified pathways differed between the two works, in both of the cases the involvement of nutrient recycling was apparent.

At the metabolome level, we did not observe any of the changes in AAs and other primary metabolites described by Moschen et al. [21]. Conversely, the same authors did not find the decrease in secondary metabolites detected in our late-senescence XRQ-derived haplotype. This could be due to different genetic mechanisms at play, but also to different protocols used to profile metabolome.

Anyway, aside from the results obtained through the global analysis of transcriptome and metabolome, the specific examination of the inverted region within the LES10.179 QTL locus provided the most relevant hint towards the bases of retarded senescence. Inversions control adaptive phenotypic variations by locally reducing recombination in many species, including sunflower [94]. In our case, the most interesting finding was that this region included *HanXRQr2_Chr10g0464431*, a homolog to the *NYC1* chlorophyll b reductase which is also a SAG [95]. *NYC1* plays a key role in chlorophyll degradation and is activated in senescence [5]. In arabidopsis and rice, *nyc1* mutants (i.e. defective in *NYC1*) show a stay-green phenotype and thick grana in chloroplasts in leaf senescence [96, 97]. Furthermore, *NYC1* is also implicated in seed maturation [98].

**Fig. 5:**
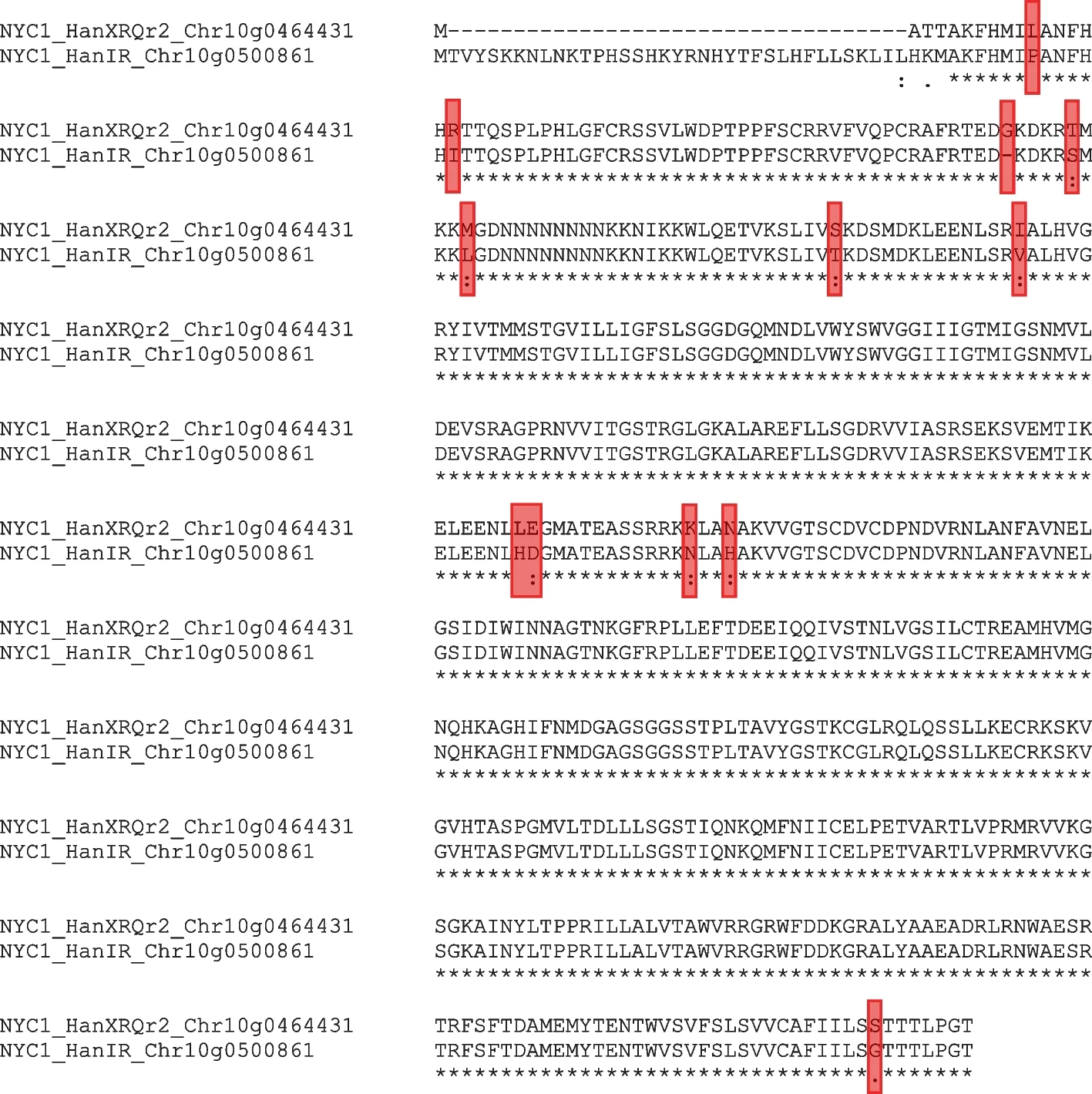
Alignment of the protein sequences of the two alleles of the *NYC1* chlorophyll b reductase sunflower homolog found in the LES10.179 QTL region. Red boxes show the positions of mutations or indels. The first 40 amino acids of the alignment have not been considered for the identification of mutations because of the poor quality of their alignment. The alignment has been performed using ClustalX 2.1

Intriguingly, Chardon et al. [2] identified a meta-QTL co-localizing with *NYC1* in three arabidopsis RIL populations and determining divergent effects on senescence and seed protein content: one allele accelerated senescence and lowered seed protein content, while the other one acted in the opposite way. Our results were consistent with those findings. Indeed, the late senescence XRQ-derived haplotype showed repression of *HanXRQr2_Chr10g0464431* (log2FC = −0.7), higher concentration of chlorophyll and increased seed protein content (Table S03, Fig. 2). Moreover, 11 nonsynonymous mutations and one presence/absence polymorphism were found between the two alleles of the gene (Fig. 5). Altogether, *NYC1* could thus be a potential candidate to explain the late senescence phenotype found in the XRQ-derived haplotype. In the light of this, the concomitant increase in chlorophyll and seed protein content may suggest a functional stay-green phenotype, even if the other yield-related traits did not differ between the two NILs (Table S03, Fig. 2). Anyway, it is also to be noted that *HanXRQr2_Chr16g0767421*, a homolog to *RbcS*, which encodes for the small subunit of Rubisco, was repressed in the XRQ-derived haplotype (Table S05B), pointing instead to a cosmetic stay-green.

Another potentially relevant gene located in the inverted region was *HanXRQr2_Chr10g0464741*, a homolog to *HCF136*, needed for the assembly of photosynthesis system II [99]. Because photosynthesis system II is degraded during senescence, and because this gene is only found in the XRQ-derived haplotype of the LES10.179 QTL, a role in maintaining stay-green could be hypothesized. Anyway, to date the involvement of *HCF136* in this context has not been demonstrated in any species.

### Cover crops affect physiological variables only up to flowering

Consistently with the work of Souques et al. [25], CCs mainly affected some morpho-physiological parameters measured during the vegetative phase and had, instead, only a limited effect on our molecular profiles, which were obtained from leaves sampled 15 days after flowering. Furthermore, the hot temperatures recorded during the experiment, and especially during the preparation of the pots, likely accelerated the mineralization of CC residues, thus reducing their long-term impact on the plants.

More in detail, the observed changes were mainly induced by vetch residues, which increased collar diameter, plant height, plant weight and ITW, while reducing oil content. An impact, although limited and difficult to characterize, concerned also metabolome, while transcriptome was not affected. Rye residues, instead, only influenced the physiological parameter ‘days to flowering’. Overall, these findings were in agreement with the green manuring effect provided by legumes [25].

## Conclusions

The overall response to drought observed in our NILs agreed with the literature, but the use of a large number of biological replicates and of a specific LC–MS protocol allowed to highlight many novel aspects, like the global decrease of secondary metabolites and the involvement of several minor compounds with potential antioxidant properties.

The results obtained from the characterization of the senescence-related LES10.179 QTL, instead, presented more limited overlaps with the previous findings obtained in sunflower [21] and pointed to the role of a homolog to the *NYC1* gene located in an inverted region of the QTL itself. *NYC1* is known to be linked to stay-green and to an increased seed protein content in other species [2], and therefore it looks promising in our context. Anyway, our data did not allow to precisely state if the stay-green phenotype we observe can be considered functional or cosmetic, and further experimental evidence will be required to confirm our hypotheses.

## Supplementary Information

Additional file 1: Document S01. Protocol used to produce the NILs used for the analyses.

Additional file 2: Document S02. Protocol used to perform synteny analysis between the two haplotypes of the LES10.179 QTL.

Additional file 3: Table S01. List of plants used in this work.

Additional file 4: Table S02. Measurements of the morpho-physiological and biochemical parameters.

Additional file 5: Table S03. Results of the ANOVA run on morpho-physiological and biochemical parameters.

Additional file 6: Table S04. Statistics of RNA-seq data and mapping results.

Additional file 7: Table S05. Differentially expressed genes obtained from the ‘WS contrast’ and from the ‘LES10.179 contrast’.

Additional file 8: Table S06. Enriched ontology terms obtained from the DEG lists corresponding to the ‘WS contrast’ and to the ‘LES10.179 contrast’.

Additional file 9: Table S07. Lists of protein-coding genes found in the two haplotypes of the LES10.179 QTL genomic region.

Additional file 10: Table S08. Annotated metabolites identified by LC–MS.

Additional file 11: Table S09. Results of the ANOVA analysis run on the annotated metabolites.

Additional file 12: Table S10. Sunflower homologs to markers of physiologically relevant hydric stress.

Additional file 13: Table S11. Sunflower homologs to *A. thaliana* senescence associated genes.

## Data Availability

Morpho-physiological and biochemical data are reported in Table S02. The FASTQ files obtained within the frame of transcriptome profiling have been submitted to the SRA repository (www.ncbi.nlm.nih.gov/sra) under the BioProject name PRJNA1321532. LC–MS data are reported in Table S08. The results of all the other analyses are reported in Tables S03-S07 and S09-S11 and in Figs. 1–5.

## Abbreviations

QTL: Quantitative trait locus
SAG: Senescence associated gene
DA: Differential analysis
DEG: Differentially expressed gene
LC-MS: Liquid chromatography coupled to mass spectrometry
DAM: Differentially abundant metabolite
ROS: Reactive oxygen species
PCA: Principal component analysis
AA: Amino acid
