# Supplementary material for: Molecular and physiological characterization of two sunflower near-isogenic lines with contrasting haplotypes for leaf senescence
Source: BMC Plant Biol. 2026 Jun 5;26:1683. doi: 10.1186/s12870-026-09110-8 (PMC13628809; doi:10.1186/s12870-026-09110-8)
Supplement: Supplementary file 1 [file 12870_2026_9110_MOESM1_ESM.docx]

**Document S01.** Protocol used to produce the NILs individuals used in our work.

1) A NAM-DRR (Nested association mapping – Double round-robin) sunflower population was created by crossing nine inbred parental lines (i.e. 2603RM, 83HR4RM, ADV, IR, OQP8, PSC8, PW3RM, RHA438 and XRQ) following a double round robin design. After four rounds of self-fertilization, 18 ‘F5’ sub-populations of recombinant inbred lines (RILs) were obtained, corresponding to 597 RILs (i.e. circa 32 RILs per family on average)
2) All the 597 RILs, as well as the 9 corresponding parental lines, were genotyped using a custom sunflower 600k SNP Axiom genotyping array (Affymetrix, Santa Clara, CA, USA). 277,901 high quality SNPs were retained for the downstream steps of analysis
3) The NAM-DRR population was then used to carry out a GWAS analysis aimed at identifying the QTL regions associated to leaf senescence in sunflower. The phenotype used for GWAS was represented by senescence progression speed, which was defined as the slope of a linear regression curve fitted on the percentage of senescent leaves measured at three time points (Bonnafous 2017). This analysis identified several significantly associated SNPs, among which eight were located on chromosome 10 in the interval between bases 179,175,456 and 180,516,611 (https://www.heliagene.org/HanXRQr2.0-SUNRISE/). This QTL was considered of particular interest because of its co-localization with the pectinesterase *HanXRQr2_Chr10g0465061* (bases 179,674,726 – 179,683,058) and was named ‘LES10.179’
4) The IR x XRQ sub-population was found to segregate for the ‘LES10.179’ QTL and was therefore screened *in silico* to find a line presenting heterozygosity at that locus. Following the strategy described by Tuinstra et al (1997), this line was used to produce an inbred family by self-fertilization of a unique ‘F6’ plant.
5) Subsequently, six KASPar markers were developed within a 500 kb range downstream and upstream *HanXRQr2_Chr10g0465061* using the context sequences of six corresponding Axiom markers.
6) The generation ‘F7’ was genotyped using the six aforementioned KASPar markers, to identify near-isogenic lines (NILs) homozygous for the XRQ or for the IR allele at the ‘LES10.179’ QTL
7) These segregating individuals, which presented a high level of homozygosity, were hereafter considered as NILs, and further multiplied to be used in our work

**References**

- Bonnafous F. **2017**. Prise en compte d’informations a priori en sélection génomique dans un dispositif d’hybrides de tournesol (*Helianthus annuus* L.), PhD thesis. Université Toulouse 3. https://theses.hal.science/tel-03934807v1
- Tuinstra MR, Ejeta G, Goldsbrough PB. **1997**. Heterogeneous inbred family (HIF) analysis: a method for developing near-isogenic lines that differ at quantitative trait loci: Theoretical and applied genetics 95, 1005–1011
