# Supplementary material for: Molecular and physiological characterization of two sunflower near-isogenic lines with contrasting haplotypes for leaf senescence
Source: BMC Plant Biol. 2026 Jun 5;26:1683. doi: 10.1186/s12870-026-09110-8 (PMC13628809; doi:10.1186/s12870-026-09110-8)
Supplement: Supplementary file 2 [file 12870_2026_9110_MOESM2_ESM.docx]

**Document S02.** Protocol used to perform synteny analysis between the XRQ-derived and the IR-derived haplotypes of the LES10.179 QTL.

1. The genes belonging to the XRQ-derived haplotype of the LES10.179 QTL locus (chromosome 10: 178,460,000 – 181,400,000) were extracted from the ‘Heliagene’ website (https://www.heliagene.org/HanXRQr2.0-SUNRISE/). A total number of 214 genes were found, including 193 protein-coding genes, 19 genes corresponding to lncRNAs and two to tRNAs
2. The python version of the ‘MCScanX’ tool (https://github.com/tanghaibao/jcvi/wiki/Mcscan-(python-version)), which is part of the JCVI python package (Tang *et al.,* 2024), was used with standard parameters to identify the homologs to XRQ genes in the IR genome and characterize the gene-level synteny between the two haplotypes of the LES10.179 QTL. In order to carry out this analysis, ‘MCScanX’ took into account only the 193 protein-coding genes of the XRQ-derived haplotype. This corresponded to 192 protein-coding genes in the IR-derived haplotype
3. To refine the comparison between the two haplotypes, all the potential presence/absence variations were manually checked by performing tBLASTn analyses. The vast majority of these proteins were either short (i.e. < 50 amino acids) or did not return any significant tBLASTn match and were therefore considered artifactual and removed. Eventually, 153 protein-coding genes in the XRQ-derived haplotype and 148 genes in the IR-derived haplotype were retained, with 19 cases of presence/absence variation
4. The final plot showing synteny patterns between the two haplotypes was created using the corresponding functions of ‘MCScanX’

**References**

Tang H, Krishnakumar V, Zeng X, Xu Z, Taranto A, Lomas JS, Zhang Y, Huang Y, Wang Y, Yim WC, Zhang J, Zhang X. JCVI: A versatile toolkit for comparative genomics analysis. iMeta. 2024 Jun 12;3(4):e211
